# Crosstalk Between Mammalian Autophagy and the Ubiquitin-Proteasome System

**DOI:** 10.3389/fcell.2018.00128

**Published:** 2018-10-02

**Authors:** Nur Mehpare Kocaturk, Devrim Gozuacik

**Affiliations:** ^1^Molecular Biology, Genetics and Bioengineering Program, Faculty of Engineering and Natural Sciences, Sabanci University, Istanbul, Turkey; ^2^Center of Excellence for Functional Surfaces and Interfaces for Nano Diagnostics (EFSUN), Sabanci University, Istanbul, Turkey; ^3^Nanotechnology Research and Application Center (SUNUM), Sabanci University, Istanbul, Turkey

**Keywords:** autophagy, UPS, proteasome, ubiquitylation, protein quality control, mitophagy, proteostasis, organelle homeostasis

## Abstract

Autophagy and the ubiquitin–proteasome system (UPS) are the two major intracellular quality control and recycling mechanisms that are responsible for cellular homeostasis in eukaryotes. Ubiquitylation is utilized as a degradation signal by both systems, yet, different mechanisms are in play. The UPS is responsible for the degradation of short-lived proteins and soluble misfolded proteins whereas autophagy eliminates long-lived proteins, insoluble protein aggregates and even whole organelles (e.g., mitochondria, peroxisomes) and intracellular parasites (e.g., bacteria). Both the UPS and selective autophagy recognize their targets through their ubiquitin tags. In addition to an indirect connection between the two systems through ubiquitylated proteins, recent data indicate the presence of connections and reciprocal regulation mechanisms between these degradation pathways. In this review, we summarize these direct and indirect interactions and crosstalks between autophagy and the UPS, and their implications for cellular stress responses and homeostasis.

## Introduction

The ubiquitin–proteasome system (UPS) and macroautophagy (hereafter referred as autophagy) are two major intracellular protein degradation pathways. Degradation of short-lived proteins through the UPS is initiated by sequential addition of ubiquitin chains to target proteins (Hershko, 1983, 2005; Finley, 2009). Polyubiquitylated proteins are then recognized by the subunits of multicatalytic protease complexes called proteasomes (Hershko and Ciechanover, 1998; Schwartz and Ciechanover, 2009).

Proteasomes are extremely efficient organelles that degrade short-lived proteins and soluble unfolded/misfolded proteins and polypeptides. On the other hand, long-lived proteins, insoluble protein aggregates (usually originating from misfolded proteins, disease-related mutant proteins) and dysfunctional organelles, such as degenerated mitochondria and peroxisomes, are eliminated by the autophagy-lysosome system (Groll and Huber, 2003, 2004; Klionsky, 2007). Autophagy is characterized by the formation of double-membrane structures termed as autophagosomes, which later on fuse with lysosomes, forming autolysosomes degrading autophagosome contents.

The UPS and autophagy are interconnected, and inhibition of one system was shown to affect the other. There is accumulating evidence in the literature about connections between the UPS and autophagy. In this review article, we will first briefly summarize the two systems, and then discuss in detail various examples of coordination and crosstalk between them. For more detailed discussion on individual systems, the readers are referred to recently published excellent review articles (Collins and Goldberg, 2017; Kwon and Ciechanover, 2017; Mizushima, 2018; Yu et al., 2018). This review article mainly focuses on the mammalian system and advances in this field. For crosstalk in other systems, such as plants, readers should check other recent and relevant reviews [for example see, (Minina et al., 2017)].

### The Ubiquitin-Proteasome System

Ubiquitylation-dependent degradation is involved in the regulation of several cellular processes, including protein quality control, transcription, cell cycle progression, DNA repair, cell stress response and apoptosis. For example during cell cycle regulation, timely progression of each phase of the cycle rely on sequential transcription and degradation of cell cycle proteins, such as cyclins (Glotzer et al., 1991; Benanti, 2012). During apoptosis, ubiquitylation leading the degradation of survivin depends on ubiquitin ligase XIAP (Arora et al., 2007; Altieri, 2010; Delgado et al., 2014).

**FIGURE 1:**
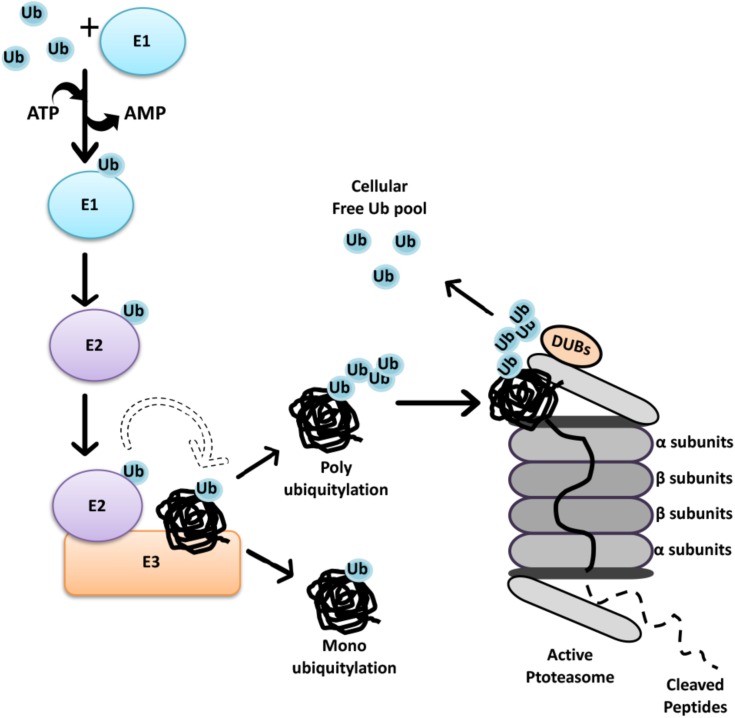
The Ubiquitin-Proteasome System. Initially through C-terminal glycine, ubiquitin is attached to a cysteine residue of an activating enzyme, E1, in an ATP-dependent manner. The active ubiquitin is then associated with a cysteine residue of an ubiquitin conjugating enzyme, E2. Finally, specificity of ubiquitin transfer is ensured by E3 ubiquitin ligase family of proteins that bind to selected protein subsets (Hershko and Ciechanover, 1998). In the case of RING finger E3 ligases, the transfer of ubiquitin is direct from E2-ubiquitin to the substrate, even if the presence of E3 is required for substrate selection. At present, 2 genes are known to encode E1 isoforms, at least 40 genes encode E2’s, and over 600 E3 ubiquitin ligases were defined in the human genome (Pickart and Eddins, 2004; Clague et al., 2015). Each E1 isoform reveals a distinct preference for different E2 enzymes, while association of E2 and E3 depend on cellular context, generating extensive combinatorial complexity.

Ubiquitylation involves the addition of the small protein ubiquitin to specific lysine residues on the target proteins. Covalent attachment of ubiquitin to protein targets occurs through a three-step mechanism involving E1 (ubiquitin-activating), E2 (ubiquitin-conjugating) and E3 (ubiquitin ligase) enzymes as summarized in **Figure 1** (Hershko and Ciechanover, 1998). At least seven lysine (K) residues in the ubiquitin protein are involved in the polyubiquitin chain formation (K6, K11, K27, K29, K33, K48, or K63). Initially, K48-linked ubiquitin chain formation was introduced as the degradation signal for proteasomal degradation. In contrast, K11 or K63 chains or single ubiquitin moieties (monoubiquitylation) were initially connected to non-proteolytic functions (Welchman et al., 2005; Behrends and Harper, 2011). However, recent reports indicate that K63-linked ubiquitin chains as well as various other chains prime substrates for autophagic elimination (Tan et al., 2008b).

The 26S proteasome is an ATP-dependent protease complex, consisting of a core complex, the 20S proteasome and a regulatory complex, the 19S proteasome cap. The 20S proteasome forms a barrel-shape structure with two end rings formed by α subunits regulating the entry of unfolded proteins, and two middle rings are composed of β subunits harboring proteolytic activity (Heinemeyer et al., 2004). Substrates must be unfolded and then guided by α subunits prior to catalytic cleavage. At the end, polypeptides are chopped into 3–25 amino acid long fragments, and further cleavage to single amino acids is carried out by peptidases (Tomkinson and Lindås, 2005) (**Figure 1**). By this way, recycling of proteins result in the generation of amino acids that are ultimately reused by cells in the synthesis of new proteins.

The 26S proteasome contains an additional 19S cap structure that further regulates the internalization of ubiquitylated substrates (Lander et al., 2012). The central part of the 19S cap consists of six AAA ATPases (Rpt1–Rpt6) forming the Rpt ring that is responsible for substrate binding and unfolding as well as substrate transfer through the channel (Collins and Goldberg, 2017). Non-ATPase proteins such as Rpn10 and Rpn13 in the 19S cap, possess ubiquitin-binding domains and therefore function as receptors for ubiquitin-labeled substrates (Finley, 2009).

Recent studies showed that ubiquitylation is a reversible phenomenon. Deubiquitinating enzymes (DUBs) are proteases that remove ubiquitin or ubiquitin-like molecules from substrates and disassemble polyubiquitin chains. DUBs regulate UPS-mediated degradation in different cellular contexts (Reyes-Turcu et al., 2009; He et al., 2016; Pinto-Fernandez and Kessler, 2016). Moreover, they play an important role in the control of available free ubiquitin pool in cells, allowing recycling and reuse of ubiquitin. Some DUBs are also responsible for processing newly synthesized ubiquitin precursors (Komander et al., 2009; Lee et al., 2011; Grou et al., 2015; Collins and Goldberg, 2017).

### Autophagy

There are three major types of autophagy: Macroautophagy, microautophagy and chaperon-mediated autophagy (CMA). In this review, we chose to focus on macroautophagy (herein autophagy). CMA and microautophagy were discussed in elsewhere (Kaushik and Cuervo, 2018; Oku and Sakai, 2018).

**FIGURE 2:**
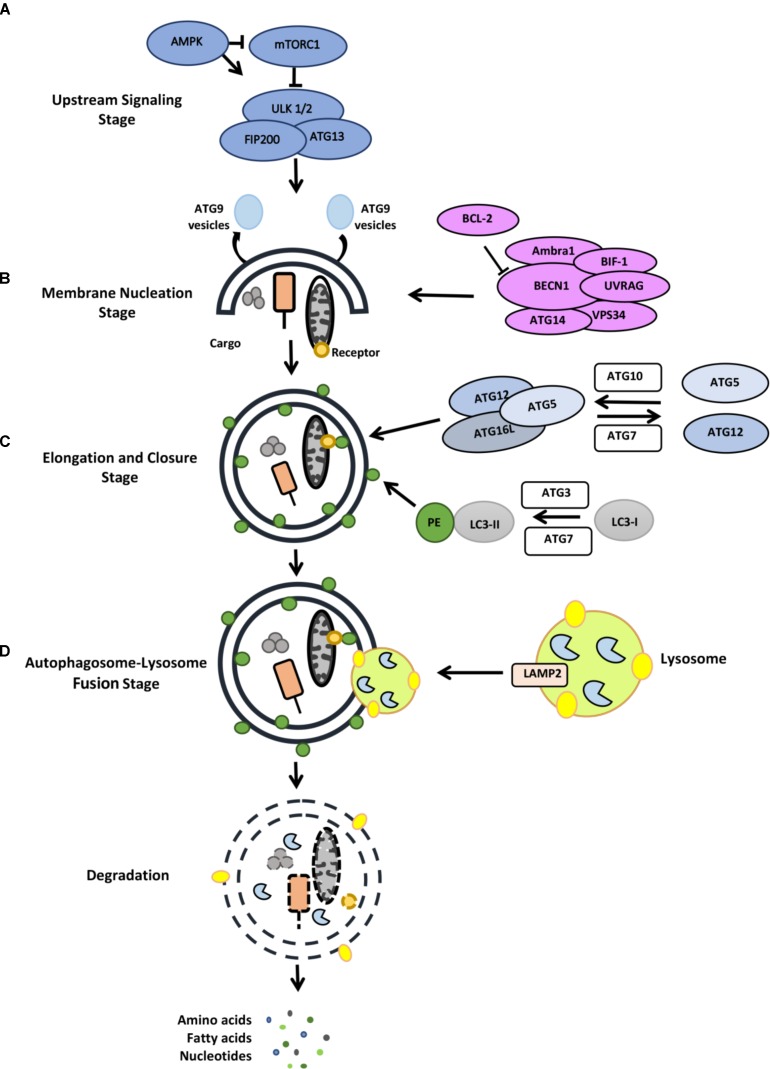
Stages of the autophagy pathway (for detail, see the text). **(A)** Upstream signaling, **(B)** membrane nucleation stage, **(C)** elongation and closure stage, **(D)** autophagosome-lysosome fusion stage.

Autophagy is characterized by the engulfment of cargo molecules by double-membrane vesicles, called autophagosomes (Klionsky, 2007; Mizushima, 2010, 2018; Lamb et al., 2013). Following closure, autophagosomes are transported by the microtubule system, leading to their fusion with late endosomes and lysosomes, forming autolysosomes. In this new compartment, sequestered cargos are degraded by the action of lysosomal hydrolases. Building blocks that are generated by hydrolysis of macromolecules (e.g., amino acids from protein degradation) are then transferred back to cytosol for reuse (**Figure 2**). Active at a basal level, autophagy is upregulated following a number of stimuli and stress conditions. Amino acid deprivation, serum starvation and growth factor deprivation, hypoxia, exposure to various chemicals and toxins might be counted among stress conditions activating autophagy.

Most autophagy inducing signals converge at the level of mTOR protein complexes (mTORC1 and mTORC2) that coordinate anabolic and catabolic processes (Sabatini, 2017; Saxton and Sabatini, 2017) (**Figure 2**). Cellular energy sensor AMPK directly regulates mTOR and therefore contributes to the regulation of the autophagic activity. Moreover, the ERK/RSK pathway, PI3K/AKT pathway, amino acid sensor RAG system as well as hypoxia are among autophagy-related pathways converging at the level of mTOR. Under normal conditions, mTORC1 limits the autophagic activity through inactivation of the ULK1/2 autophagy complex. mTORC1-dependent phosphorylation of ULK1 and Atg13 (Hosokawa et al., 2009) result in the inactivation of ULK1/2 complex and down regulation of autophagy. Under stress, mTORC1 is inhibited and ULK1/2 complex dephosphorylated. ULK1/2 then phosphorylates itself, Atg13 and FIP200 and activate autophagy.

A class III phosphatidylinositol 3-kinase (PI3K) complex, including the lipid kinase VPS34 and the regulatory protein Beclin1, controls the membrane nucleation stage and initial phagophore formation. Phosphatidylinositol 3-phosphate (PtdIns3P) that is generated by PI3K activity serves as a landing pad for autophagy-related proteins containing PI3P-binding domains (e.g., FYVE-domains). Among them WIPI1-4 and DFCP1 were involved in the formation of a membrane structure called omegasome or cradle, a structure that creates a platform for the elongation of autophagosome precursor isolation membranes (Mauthe et al., 2011; Mercer et al., 2018).

Elongation of the isolation membrane depends on two ubiquitin-like conjugation systems. In the first system, autophagy-related gene 12 (ATG12) is covalently conjugated to the ATG5 protein through the action of ATG7 (E1-like) and ATG10 (E2-like) proteins. Then, recruitment of the ATG16L1 protein to ATG12-5 dimer results in the formation of a larger complex. Then forming ATG12-5-16L1 oligomers serve as E3 ligases that conjugate lipid molecules (such as phosphatidylethanolamine) to ATG8 orthologs MAP1LC3, GATE16, GABARAP (Mizushima et al., 2011; Shpilka et al., 2011; Tsuboyama et al., 2016). Lipid-conjugated ATG8 proteins are required for the elongation, expansion and closure of autophagosome membranes (Nakatogawa et al., 2007).

In order to acquire lytic capacity, autophagosomes fuse with late endosomes or lysosomes. In mammalian cells, fusion requires lysosomal integral membrane protein LAMP-2, several SNARE proteins (e.g., STX17 and WAMP8) and RAB proteins (e.g., RAB5 and RAB7) (Tanaka et al., 2000; Jager, 2004). Following fusion of the outer membrane of autophagosomes, materials contained in the inner membrane are degraded by the action of lysosomal hydrolases (Tanida et al., 2004). Building blocks (e.g., amino acids, fatty acids etc.) are then transported back to cytosol for reuse in the metabolic processes of the cells.

Autophagic vesicles engulf targets such as portions of cytoplasm and various cytoplasmic components in a non-selective manner. On the other hand, several selective forms of autophagy have been described (Kraft et al., 2010; Anding and Baehrecke, 2017). In most cases, ubiquitylation of the cargo constitutes a key step in the chain of events leading to its autophagic removal (Kirkin et al., 2009; Rogov et al., 2014). Selective targets include mitochondria (Okamoto et al., 2009), peroxisomes (Till et al., 2012), lysosomes (Hung et al., 2013), endoplasmic reticulum (ER) (Khaminets et al., 2015), ribosomes (An and Harper, 2018), cytoplasmic protein aggregates (Lamark and Johansen, 2012), pathogenic intracellular invaders (Wileman, 2013) and even certain free proteins and RNAs (Huang et al., 2015) were shown to be targets of selective autophagy. By this way, cells control number of the organelles, eliminate dysfunctional components and get rid of potentially harmful aggregates and invaders.

Selectivity is ensured by target-specific autophagy receptors that form a bridge between the ubiquitylated cargo and LC3 component of autophagic membranes. Selective autophagy relies on the recognition and binding capacity of autophagy receptors to various types of cargo, including mitochondria (OPTN, NDP52, Tax1BP1, NIX, FUNDC1) (Novak et al., 2010; Sarraf et al., 2013; Wong and Holzbaur, 2014; Lazarou et al., 2015; Chen et al., 2016), peroxisomes (NBR1) (Deosaran et al., 2013), lysosomes (galectin-3) (Hasegawa et al., 2015), ER (FAM134B, SEC62, RTN3, and CCPG1) (Khaminets et al., 2015; Fumagalli et al., 2016; Grumati et al., 2017; Smith et al., 2018) and intracellular ubiquitylated aggregates (p62, NBR1, OPTN, TOLLIP receptors) (Pankiv et al., 2007; Kirkin et al., 2009; Korac et al., 2013; Lu et al., 2014), bacterial invaders (p62, OPTN, NDP52 receptors) (Thurston, 2009; Zheng et al., 2009; Wild et al., 2011). LC3-interacting region (LIR) is the common motif which allows autophagy receptors to bind lipidated LC3. On the other hand, ubiquitin-associated domain (UBA domain) on autophagy receptors are responsible for the recognition of ubiquitin decorated cargos (Khaminets et al., 2016). Cargos that are wrapped and packed in autophagosomes are then ready for delivery and degradation in lysosomes.

## The UPS-Autophagy Connection

The UPS and autophagy are the two major and evolutionarily conserved degradation and recycling systems in eukaryotes. Although their activities are not interdependent, recent studies show that connections and crosstalks exist between the two systems. Mitophagy constitutes a prominent example connecting these two degradative systems, yet several other examples exist. In this section, we will summarize biological events involving autophagy and the UPS, and discuss molecular details of the crosstalk mechanisms.

### Compensation Between the Two Degradative Pathways

**FIGURE 3:**
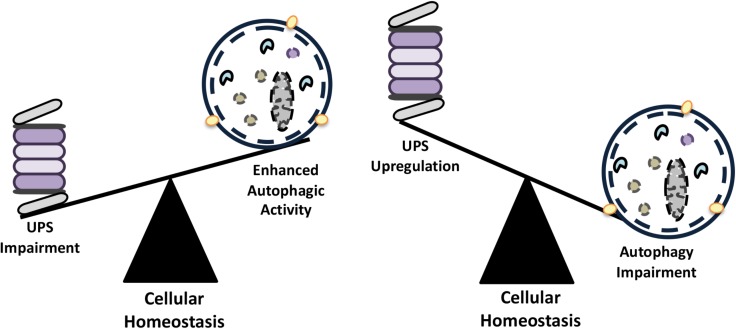
The compensatory balance between the activities of autophagy and the UPS in order to maintain cellular homeostasis.

Initial observations about functional connections between the UPS and autophagy systems revealed that inhibition of one led to a compensatory upregulation of the other system. In order to maintain homeostasis, cellular materials that accumulate following inhibition of one degradative system needs to be cleared, at least in part, by the other system (**Figure 3**). Here, we will give examples of scenarios where these compensation mechanisms are operational.

Inhibition of the UPS using various compounds (e.g., MG132, bortezomib, lactacystine etc.) (Wu et al., 2008; Selimovic et al., 2013; Fan et al., 2018) or by genetic approaches (Demishtein et al., 2017) resulted in the upregulation of the autophagic activity in cells (**Figure 3**). For example, inhibition of proteasomal activity by the proteasome inhibitor and chemotherapy agent bortezomib led to an increase in the expression of autophagy genes ATG5 and ATG7, and induced autophagy. In fact, autophagy gene upregulation depended on an ER stress-dependent pathway that involved eukaryotic translation initiation factor-2 alpha (eIF2α) phosphorylation (Zhu et al., 2010). In another study, proteasome inhibition was associated with an increase in p62 and GABARAPL1 levels by Nrf1-dependent and -independent pathways prior to autophagy activation (Sha et al., 2018). In other contexts, MG132-mediated proteasome inhibition resulted in a decrease in cell proliferation, cell cycle arrest at G2/M phase and stimulation of autophagy through upregulation of Beclin1 and LC3 (Ge et al., 2009).

Autophagy induction following proteasome inhibition correlated with AMPK activation as well. A number of studies provided evidence that proteasomal inhibition is sensed by both AMPK and mTORC1, two major regulators of autophagy. For instance, in macrophages, epitelial and endothelial cells, proteasome inhibition using chemicals resulted in the activation of AMPK (Xu et al., 2012; Jiang et al., 2015). In some other cancer cell types, CaMKKβ and glycogen synthase kinase-3β (GSK-3β) were identified as upstream regulators of AMPK activation, proteasome inhibition was linked to a decrease in GSK-3β activity and to the activation of AMPK and autophagy (Sun et al., 2016). On the other hand, Torin-1- or rapamycin-mediated inhibition of mTOR stimulated long-lived protein degradation through activation of both UPS and autophagy (Zhao et al., 2015; Zhao and Goldberg, 2016). In retinal pigment epithelial cells, inhibition of proteasome by lactacystin and epoxomicin was shown to block the AKT-mTOR pathway and induce autophagy (Tang et al., 2014). SiRNA-mediated knockdown of Psmb7 gene coding for the proteasome β2 subunit, resulted in enhanced autophagic activity, and it was linked the mTOR activation status of cultured cardiomyocytes (Kyrychenko et al., 2013).

Similarly, impairment of autophagy correlated with the activation of the UPS. In colon cancer cells, chemical inhibition of autophagy and small RNA mediated knock down of ATG genes resulted in the upregulation of proteasomal subunit levels, including the catalytic proteasome β5 subunit, PSMB5 and led to increased UPS activity (Wang et al., 2013). In another study, 3-MA-mediated autophagy inhibition in cultured neonatal rat ventricular myocytes (NRVMs) increased chymotrypsin-like activity of proteasomes (Tannous et al., 2008).

Since proteasomes were identified as autophagic degradation targets (proteaphagy), enhanced proteasome peptidase activity following autophagy inhibition might be associated with the accumulation of proteasomes (Cuervo et al., 1995; Marshall et al., 2015). Yet in several cases, autophagy inhibition correlated with the accumulation of ubiquitylated proteins. For instance in independent studies with ATG5 or ATG7 knockout mice, accumulation of ubiquitylated conjugates were observed, especially in the brain and the liver of the animals (Komatsu et al., 2005, 2006; Hara et al., 2006; Riley et al., 2010). Similar results were observed in other animal models such as *Drosophila* (Nezis et al., 2008). In line with these data, inhibition of autophagy through siRNA-mediated knockdown of ATG7 and ATG12 in HeLa cells resulted in the impairment of UPS, accumulation of ubiquitylated proteins as well as other important UPS substrates, including p53 and β-catenine (Korolchuk et al., 2009a). In above-cited papers, autophagy impairment followed by the autophagy receptor p62 accumulation in cells, and played a key role in the observed UPS defects.

Ubiquitylation was proposed to be a common component that directs substrates to the proper degradation system and even contribute to the UPS-autophagy crosstalk (Korolchuk et al., 2010; Dikic, 2017). According to this view, proteins that are predominantly linked to K48-based ubiquitin chains are generally directed for degradation through UPS. Conversely, aggregates that are linked to K63-based ubiquitin chains are directed for autophagic degradation. P62 binding capacity was introduced as the critical step in the choice between the UPS and autophagy. Although, p62 is able to attach both K48- and K63-linked ubiquitin chains through its UBA domain, binding affinity of the protein for K63-linked chains seems to be higher (Long et al., 2008; Tan et al., 2008a; Wooten et al., 2008). Due to this dual ubiquitin binding ability, p62 might show UPS inhibitory effects in some contexts. A competition between p62 and p97/VCP (a ubiquitin binding ER-associated degradation protein) determined the fate of ubiquitylated proteins in cells (Korolchuk et al., 2009a,b). Over expression of p97/VCP protein prevented binding of p62 to ubiquitylated substrates, and directed them for degradation by the UPS. On the other hand, accumulation of p62 following autophagy inhibition led to the sequestration of proteins that were otherwise p97/VCP targets.

In summary, in the case of a defect in one of the two degradation systems, the other system is upregulated in order to eliminate ubiquitylated protein substrates. Yet, compensation does not always work and its success largely depends on cell types, cellular and environmental conditions and target protein load.

#### Interplay Between the UPS-Autophagy in the Selective Clearance of Cytosolic Proteins

Function of proteins depend on their proper folding and 3D structures. Various insults, including heat shock, organellar stress, oxidative stress etc., might lead to the accumulation of unfolded or misfolded proteins. Moreover several disease-related mutations were associated with folding problems. Failure to refold result in dysfunctional or malfunctional, hence toxic protein accumulations, activation of stress and even cell death pathways. In order to control toxic protein accumulations, an active process of protein aggregate formation comes into play. Additionally some proteins, including mutant proteins are already prone to form aggregates. Selective clearance of most cytosolic proteins require ubiquitylation. Depending on their solubility, ubiquitylated proteins and protein aggregates are then cleared by the UPS or autophagy.

Soluble fractions of proteins with a folding problem are recognized by the chaperone machinery and directed to the UPS for degradation. Hsp70 and Hsp90 chaperone interactor CHIP was identified as one of the E3 ligases that are responsible for K48-linked ubiquitin chain addition to unfolded/misfolded proteins. BAG family proteins, especially BAG1, interact with the Hsp70 complex and induce proteasomal degradation of client proteins.

**FIGURE 4:**
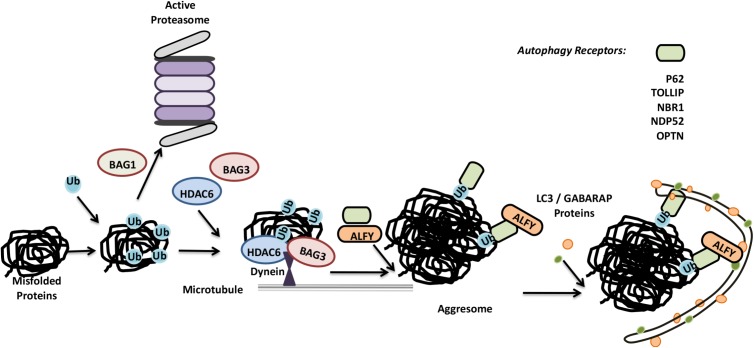
Misfolded proteins can be eliminated by both the UPS and autophagy system. Misfolded proteins are ubiquitylated and based on the differences in ubiquitin linkages and ubiquitin binding proteins, they are directed for proteasomal degradation or further accumulated in aggresomes. Aggresomes are selectively cleared by autophagy.

On the other hand, clearance of insoluble aggregate-prone proteins require formation of aggresomes. Ubiquitylation by a number of different E3 ligases, including CHIP, Parkin, HRD1 and TRIM50 prime aggregate-prone proteins (Olzmann et al., 2007; Mishra et al., 2009; Zhang and Qian, 2011; Mao et al., 2017). HDAC6 is another protein that plays a key role in the process of aggresome formation. HDAC6 was shown to provide the link between K63-based ubiquitylated aggregates and microtubule motor protein dynein (Matthias et al., 2008; Olzmann et al., 2007). Then, dynein-mediated mechanism direct the aggregates toward microtubule organizing centers (MTOCs), resulting in their piling of as aggresomes (Johnston et al., 1998; Kopito, 2000) (**Figure 4**). Following aggresome formation, direct interaction of adaptor proteins p62 and NBR1 with ubiquitylated aggregates result in their delivery to autophagosomes (Ichimura et al., 2008; Lamark and Johansen, 2012). Another autophagy-related protein, ALFY, was also identified as a player in the selective autophagy and degradation of aggresomes (Clausen et al., 2010; Filimonenko et al., 2010).

An alternative pathway for aggresome formation require Hsp70 partner proteins BAG3 and CHIP (Zhang and Qian, 2011). Similar to HDAC6, BAG3 binds to dynein, and this directs Hsp70 substrates to aggresomes. However, BAG3-dependent aggresome formation was not dependent on the ubiquitylation of substrates as in the case of HDAC6, and CHIP E3 ligase activity was dispensible (Gamerdinger et al., 2011; Zhang and Qian, 2011). Yet, E3 ligases such as CHIP were required for BAG3-dependent aggresome clearance by autophagy (Klimek et al., 2017).

### Proteolytic Degradation of the UPS or Autophagy Components as a Mutual Control Mechanism

Until so far, we focused on the UPS and autophagy as complementary but independent mechanisms. However, there are cases where components of one system were reported to be a proteolytic target of the other system. For example, a number of autophagy proteins were regulated through degradation by the UPS. On the other hand, even the whole proteasomes were shown be selective targets of autophagic degradation. Here, we will give examples of how mutual regulation through proteolysis contributes to the crosstalk and the interplay between the two systems.

#### Control of the UPS by the Autophagic Activity

**FIGURE 5:**
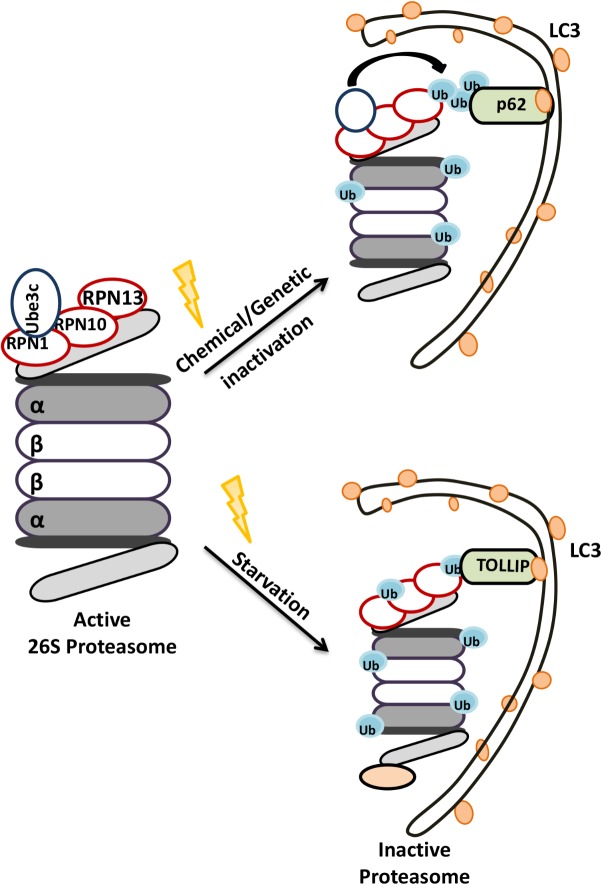
Schematic representation of the selective degradation of proteasomes by autophagy. Upon starvation and functional defects proteasomes become ubiquitylated and degraded by autophagic machinery.

Early studies indicated that proteasomes could be degraded in lysosomes (Cuervo et al., 1995). Later on, plant studies revealed that lysosomal degradation of 26S proteasomes occurred by a specific form of selective autophagy, proteaphagy (Marshall et al., 2015). RPN10 protein was introduced as an ATG8 interacting plant proteaphagy receptor. Unlike the plant protein, yeast and mammalian RPN10 failed to interact with ATG8/LC3. Instead, Cue5 protein in the yeast and its human ortholog TOLLIP, were introduced as selective receptors regulating proteasome clearance by autophagy (Lu et al., 2014). Moreover, p62 was also described as another proteaphagy receptor (Cohen-kaplan et al., 2016). For example, in mammals, amino acid starvation significantly upregulated ubiquitylation of 19S proteasome cap components RPN1, RPN10, RPN13, and led to their p62-mediated recruitment to autophagosomes (Cohen-kaplan et al., 2016) (**Figure 5**). Interestingly during carbon or nitrogen starvation, plant and yeast proteasomes were shown to localize in proteasomal storage granules (PSGs), protecting them from autophagic degradation during stress (Peters et al., 2016; Marshall and Vierstra, 2018). Whether similar mechanisms exist in the mammals is currently an open question. These observations underline the importance of selective degradation of proteasome by autophagy in the control of proteasome numbers as well as overall UPS and lytic activity in cells.

#### Control of Autophagy Components by the UPS

Modulation of the half-life of some proteins in the autophagy pathway by the UPS serves as a means to control cellular autophagic activity. For instance, LC3 protein was shown to be processed in a stepwise manner by the 20S proteasome, a process that was inhibited by p62 binding (Gao et al., 2010). On the other hand, E3 ligase NEDD4-mediated K11-linked ubiquitylation of Beclin1 prevented its binding to the lipid kinase VPS34, and led to its degradation (Platta et al., 2012). Another E3 ligase, RNF216 ubiquitylated Beclin1 adding K48-linked ubiquitin chains on the protein (Xu et al., 2014). Beclin1 ubiquitylation resulted in autophagy blockage in both cases. Conversely, reversal of Beclin1 ubiquitylation by the DUB protein USP19 stabilized the protein under starvation conditions and promoted autophagy (Jin et al., 2016). USP10 and USP13 as well as USP9X were characterized as other DUBs that regulated autophagy through control of Beclin1 stability (Liu et al., 2011; Jin et al., 2016).

Beclin1 is not the only autophagy protein that is targeted by the UPS in a controlled manner. G-protein-coupled receptor (GPCR) ligands and agonists were reported to regulate cellular Atg14L levels, and therefore autophagy, through ZBTB16-mediated ubiquitylation of the protein (Zhang T. et al., 2015). Serum starvation increased GSK3β-mediated phosphorylation of ZBTB16, leading to its degradation. Under these conditions, stabilization of Atg14L restored of autophagy. AMBRA1 is another UPS-controlled autophagy protein. Cullin-4 was identified as an E3 ligase that was responsible for the ubiquitylation of AMBRA1, dooming it for degradation under nutrient-rich conditions where autophagy should be inhibited (Antonioli et al., 2014). The PI3K complex subunit p85b is another example. Ubiquitylation of this autophagy signaling component by the E3 ligase SKP1 led to a decrease in its cellular levels and stimulated autophagic activity (Kuchay et al., 2013).

Ubiquitylation of some autophagy proteins did not result in their immediate proteasomal degradation, yet the post-translational modification provided an extra layer of control for the autophagy pathway. For instance, autophagy receptor OPTN was ubiquitylated as a target of the E3 ligase HACE1, and K48-linked ubiquitylation regulated the interaction of the protein with p62 (Liu Z. et al., 2014). TRAF6, a central E3 ligase of the NF-κB pathway, participated controlled ULK1 activity through K63-linked ubiquitylation. Under nutrient-rich conditions, mTOR phosphorylated AMBRA1 leading to its inactivation. When nutrients were limiting, mTOR inhibition resulted in AMBRA1 dephosphorylation and increased the interaction of the protein with TRAF6. This event facilitated ULK1 ubiquitylation by TRAF6 (Nazio et al., 2013). Ubiquitylation of ULK1 resulted in the stabilization of the protein, controlled its dimerization and regulated its kinase activity. Another ubiquitin-dependent regulation mechanism involved AMBRA1-Cullin-5 interaction in the regulation of mTOR complex component DEPTOR (Antonioli et al., 2014). Above-mentioned AMBRA1-Cullin-4 complex dissociated under autophagy-inducing conditions, allowing AMBRA1 to bind another E3 ligase, Cullin-5. This newly formed complex was shown to stabilize DEPTOR and induce mTOR inactivation, providing a negative feed-back loop in the control of autophagy (Antonioli et al., 2014).

In another study, TLR4 signaling triggered autophagy through Beclin1 ubiquitylation and stabilization. TLR4-associated TRAF6 protein was identified as the E3 ligase responsible for K63-linked ubiquitylation of Beclin1 at its BH3 domain. This modification blocked inhibitory BCL-2 binding to the protein, and free Beclin1 could activate autophagy (Shi and Kehrl, 2010). On the other hand, the deubiquitinating enzyme A20 reversed TRAF6-mediated ubiquitylation of Beclin1, resulting in autophagy inhibition (Shi and Kehrl, 2010). Another K63-linked ubiquitylation event on Beclin1 was promoted by AMBRA1 protein. In the same context, the WASH protein interacted with Beclin1, blocked AMBRA1-mediated Beclin1 ubiquitylation, and suppressed autophagy (Xia et al., 2013).

LC3 and p62 were also subjected to regulatory ubiquitylation. NEDD4 was identified as the E3 ligase in these reactions. NEDD4 was reported to interact with LC3 (Sun et al., 2017) and p62 (Lin et al., 2017), and LC3 binding to NEDD4 stimulated its ubiquitin ligase activity on the p62 protein (Sun et al., 2017). Moreover, NEDD4 deficient cells exhibited aberrant p62 containing inclusions, indicating the defect in aggresome clearance (Lin et al., 2017). Hence, NEDD4 is important for the regulation of p62 function and autophagy.

### Xenophagy: Removal of Intracellular Invaders

**FIGURE 6:**
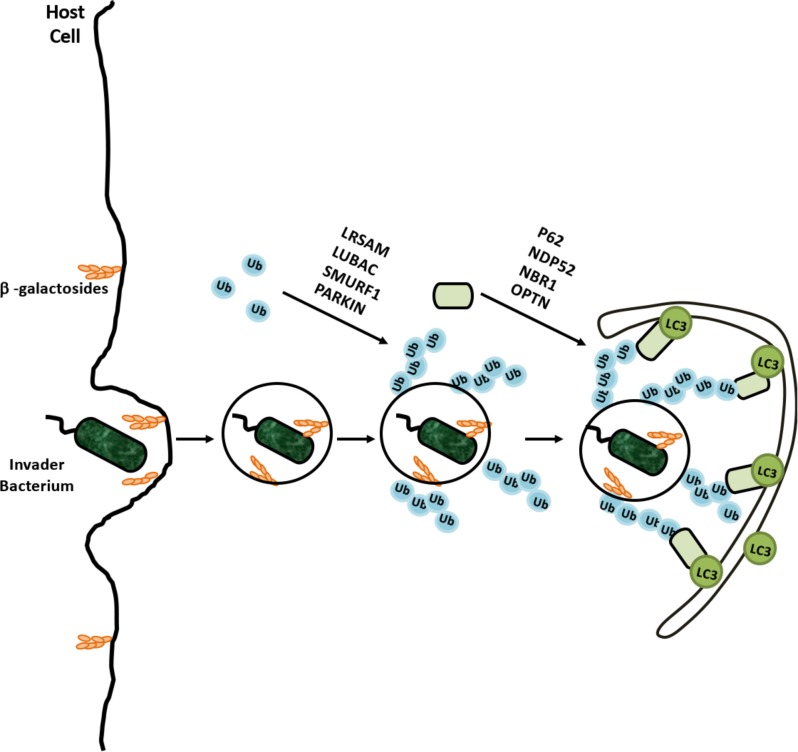
Selective degradation of invaders by xenophagy is example of coregulation of the UPS and autophagy. Cellular degradation of invading bacterium was ubiquilated by various E3 ligases and recognized by adaptor proteins for recruitment autophagic membranes around bacterium.

Another essential function of autophagy is the clearance of intracellular pathogens. This special form of autophagy, called xenophagy, is a result of a cooperation between the ubiquitylation machinery and the autophagy pathway. Pathogens such as *Streptococcus pyogenes, Mycobacterium tuberculosis, Listeria monocytogenes*, and *Shigella flexneri* were identified as autophagy targets (Gutierrez et al., 2004; Kirkegaard et al., 2004; Ogawa et al., 2005). As a form of selective autophagy, xenophagy involves cargo labeling with ubiquitin, followed by the recognition by autophagy receptors (**Figure 6**). K48- and K63-linked and linear M1-linked ubiquitin chains were shown to mediate recognition of different pathogens by the xenophagy machinery (Collins et al., 2009; Randow and Youle, 2014).

Ubiquitylation frequently occurs on various cell penetrating parasites as well as on disrupted endosomes, providing an “eat me” signal for xenophagy. For example, *Salmonella enterica* serovar Typhimurium was heavily ubiquitylated in mammalian cells, and activation of xenophagy restricted intracellular bacteria numbers (Birmingham et al., 2006). Recent studies showed that, bacterial outer membrane-associated and integral membrane proteins were targets of ubiquitylation (Fiskin et al., 2016). A number of E3 ligases were involved in xenophagy, including Parkin, RNF166, ARIH1, HOIP, and LRSAM1 (Huett et al., 2012; Manzanillo et al., 2013; Heath et al., 2016; Franco et al., 2017; Lobato-Márquez and Mostowy, 2017).

For example, both K48- and K63-linked ubiquitylation were observed on Mycobacterium, and Parkin was identified as the E3 ligase catalyzing the K63-linked ubiquitylation (Collins et al., 2009; Manzanillo et al., 2013). Moreover endosome-free areas on the intracellular *Salmonella* Typhimurium contained a directly attached ubiquitin coat, and addition of linear M1-linked ubiquitin chains by the E3 ligase HOIP of the LUBAC on these ubiquitins contributed to the autophagy of the intracellular parasite (Noad et al., 2017). Xenophagy receptors that were described to date include p62, OPTN, NDP52, and NBR1 (Thurston, 2009; Zheng et al., 2009; Wild et al., 2011). These receptors were reported to bind pathogen- and/or endosome-associated ubiquitin, and directing the selective targets to autophagic membranes (Wild et al., 2011; Richter et al., 2016).

The interplay between ubiquitylation and autophagy achieves the important task of keeping host cells pathogen-free and providing an intracellular innate immune defense mechanism against invaders. In some reports, ubiquitylated bacteria were found to be surrounded by proteasomes as well (Perrin et al., 2004) and proteasomal activity might also be required for efficient killing of intracellular parasites (Iovino et al., 2014). Whether in the elimination of invading organisms, the crosstalk between the UPS and autophagy systems goes beyond ubiquitylation, needs further consideration. As discussed below, cellular mechanisms controlling commensal-turned ancient intracellular microorganisms, namely mitochondria, indeed rely on the function of both the UPS and autophagy.

### Mitophagy: Mitochondrial Turnover

Mitochondria are vital organelles that form an intracellular dynamic network in the cytosol of eukaryotic cells. Through fusion and fission, they are constantly made and destroyed. Under steady state conditions, mitochondria might be eliminated by basal in a non-selective manner. On the other hand, elimination of damaged, dysfunctional or superfluous mitochondria requires a selective form of autophagy called mitophagy (Lemasters, 2005). Programmed elimination of mitochondria during development and differentiation (e.g., reticulocyte maturation to erythrocyte, in oocytes after fertilization, during lens formation in the eye) also relies on mitophagy (Schweers et al., 2007; Song et al., 2016; Esteban-Martínez et al., 2017). Recent studies showed that mitophagy is a biological phenomenon that involves both the UPS and autophagy. In this section, we will discuss mechanisms of mitophagy, and analyze connections between the UPS and autophagy in this context.

#### PINK1/Parkin-Dependent Mitophagy

**FIGURE 7:**
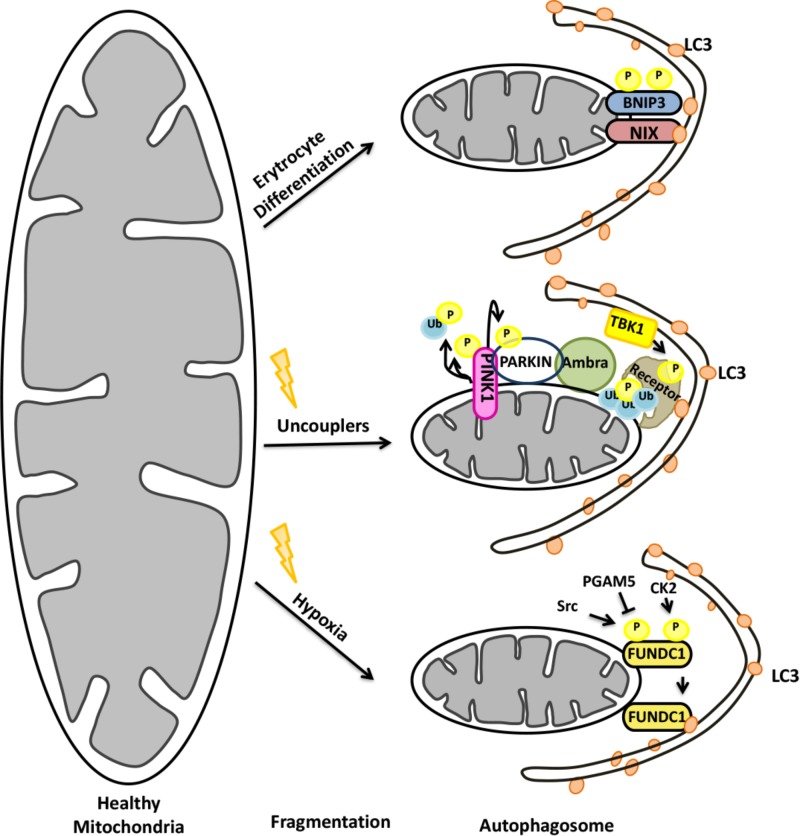
Mitochondrial elimination by autophagy requires the activity of both the UPS and autophagy.

Depending on the E3 ligase that ubiquitylates proteins on mitochondria, mitophagy can be divided into two major forms: Parkin-dependent and Parkin-independent mitophagy. The E3 ligase Parkin was first characterized as the product of the gene PARK2, mutations of which were linked to early-onset of Parkinson’s Disease. Strikingly, Parkin recruitment to mitochondria was found to be necessary for mitophagy (Narendra et al., 2008). Further studies showed that Parkin, together with another familiar Parkinson’s Disease-associated gene, PINK1 (PARK7), was responsible for priming mitochondria for autophagic degradation (**Figure 7**).

Under normal conditions, after being synthesized as precursor in the cytoplasm, PINK1 was imported to mitochondria by its N-terminal mitochondria targeting sequence (MTS). Then, PINK1 was post-translationally modified within mitochondria by resident proteases: MPP and PARL (Jin et al., 2010; Deas et al., 2011). Cleavage by PARL resulted in destabilization of the protein and its degradation by cytoplasmic proteasomes (Yamano and Youle, 2013). Under mitochondrial stress however, PINK1 cleavage did not occur and the protein accumulated on the outer mitochondrial membrane (OMM) (Lazarou et al., 2012; Hasson et al., 2013). Recruitment of cytoplasmic E3 ligase Parkin onto mitochondria required stabilization and the kinase activity of the PINK1 protein (Lazarou et al., 2012). Parkin itself was a substrate of PINK1 (Kondapalli et al., 2012; Shiba-Fukushima et al., 2012). Phosphorylation of Parkin by PINK1 resulted in a conformational change overcoming an autoinhibition, and stimulated its E3 ligase activity (Kondapalli et al., 2012; Shiba-Fukushima et al., 2012; Trempe et al., 2013; Wauer and Komander, 2013). Interestingly, PINK1 was shown to phosphorylate ubiquitin molecules on mitochondrial resident proteins as well. Ubiquitin phosphorylation correlated with an increase in the amount of mitochondria-localized Parkin, providing a feed-forward mechanism of Parkin recruitment (Kane et al., 2014; Kazlauskaite et al., 2014; Koyano et al., 2014; Shiba-Fukushima et al., 2014).

Several proteins on the mitochondrial outer membrane were identified as Parkin ubiquitylation substrates. The list includes VDAC, TOM proteins, mitofusins etc (Sarraf et al., 2013). Following ubiquitylation some of these targets were shown to be degraded by the proteasome (e.g., mitofusins) and some were not (e.g., VDAC). Degradation of proteins related to mitochondrial integrity promoted fission events that facilitate engulfment of mitochondrial portions by autophagosomes, whereas proteins that are not degraded upon ubiquitylation rather contributed to mitochondrial rearrangements (e.g., aggregation).

The UPS activity was a prerequisite in the preparation of mitochondria for autophagy. Ubiquitylation of mitochondrial targets preceeded the recruitment of the autophagic machinery onto mitochondria (Yoshii et al., 2011). Selective autophagy receptors were shown to bind ubiquitin-labeled proteins on mitochondria and recruit ATG8/LC3 proteins for mitophagy. Serial knock out of putative autophagy receptors showed that NDP52, optineurin (OPTN) and TAX1BP1 were functional mitophagy receptors, and a triple knockout of these proteins completely blocked mitophagy (Lazarou et al., 2015; Shi J. et al., 2015). On the other hand, the autophagy receptor p62 was essential for clustering of damaged mitochondria in perinuclear region of the cells, but not for mitophagy (Narendra et al., 2010; Okatsu et al., 2010).

Ubiquitin modifications on mitochondria might be reversed by the action of DUB proteins. Several DUBs were identified as positive or negative regulators of mitophagy (Dikic and Bremm, 2014; Wang et al., 2015). For example, deubiquitylation of mitochondrial targets by USP15, USP30, and USP35 prevented further progression of mitophagy in a number of cell lines and experimental models (Bingol et al., 2014; Cornelissen et al., 2014; Wang et al., 2015). DUB-mediated deubiquitylation of targets decreased Parkin recruitment onto mitochondria as well (Bingol et al., 2014). USP8-mediated removal of K6-linked ubiquitin chains from Parkin itself affected recruitment of the protein onto mitochondria and therefore mitophagy (Durcan et al., 2014; Durcan and Fon, 2015).

#### Parkin-Independent Mitophagy

Expression of Parkin is restricted to a few cell types, including dopaminergic neurons. Consequently, Parkin-null animals showed prominent mitophagy defects only in selected brain regions (Lee et al., 2018). Therefore in other cell types and tissues, mitophagy has to proceed in a Parkin-independent manner. Alternative E3 ligases were found to play a role in mitophagy in these contexts.

Mulan (MUL1) is an E3 ubiquitin ligase that resided on the OMM, and it was shown to play a role in Parkin-independent mitophagy in different model organisms, including *Caenorhabditis elegans, Drosophila* and mammals (Ambivero et al., 2014; Yun et al., 2014). Mulan stabilized DRP1, led to degradation of MFN2, and interacted with ATG8 family member protein GABARAP (Braschi et al., 2009; Ambivero et al., 2014). Another E3 ligase that was associated with mitophagy was GP78 (Christianson et al., 2012). Over expression of GP78 induced MFN1 and 2 ubiquitylation and degradation, that was followed by mitochondrial fragmentation and mitophagy in cells lacking Parkin (Fu et al., 2013). Synphilin-1-dependent recruitment of the E3 ligase Siah1 to mitochondria resulted in mitochondrial protein ubiquitylation and mitophagy in a PINK1-dependent but Parkin-independent manner (Szargel et al., 2015). Conversely, another OMM E3 ligase, MITOL (MARCH5), was reported to ubiquitylate FIS1, DRP1 (Yonashiro et al., 2006) and MFN2 (Nakamura et al., 2006), yet inhibited hypoxia-induced and Parkin-independent mitophagy through ubiquitylation and degradation of FUNDC1 (Chen et al., 2017). All these findings underline the fact that mitophagy might proceed in cells which do not express Parkin. Further studies are required to unravel the molecular mechanisms of Parkin-independent mitophagy in different tissues and cell types, and reveal the details of the crosstalk between the UPS and autophagy under these conditions.

#### A Special Type of Mitophagy During Reticulocyte Maturation

During differentiation, in order to increase their capacity to load hemoglobin-bound oxygen, reticulocytes lose their organelles, including mitochondria, and become mature red blood cells (Dzierzak and Philipsen, 2013). During this process, a protein called NIX (also known as BNIP3L) is upregulated (Aerbajinai et al., 2003). NIX is a C-terminally anchored outer mitochondrial membrane (OMM) protein that contains a LC3-interacting region (LIR) at its cytoplasmic N-terminal part. Through its LIR domain, NIX interacted with LC3, enabling engulfment of mitochondria by autophagosomes in reticulocytes (Novak et al., 2010). Characterization of NIX-deficient mice showed that, NIX-deficient Erythrocytes failed to eliminate their mitochondria revealing a critical role for NIX in mitophagy (Schweers et al., 2007; Sandoval et al., 2008) (**Figure 7**). Although NIX-dependent mitophagy was predominantly studied in reticulocytes, NIX-dependent mitophagy might be important for other cell types as well [for example, see (Esteban-Martínez et al., 2017)].

A role for the UPS in NIX/BNIP3L-dependent mitophagy was revealed. NIX/BNIP3L was discovered to be ubiquitylated through a PINK1/Parkin-dependent mechanism. Ubiquitylated NIX/BNIP3L colocalized with selective autophagy receptors, and the process was necessary for mitochondrial stress-induced mitophagy (Ding et al., 2010; Gao et al., 2015; Palikaras et al., 2015). Therefore, the role of NIX/BNIP3L seems to be more general than previously thought and beyond the developmental context, and stress-induced mitochondrial elimination by autophagy might also require NIX/BNIP3L in different cell and organism types.

### Pexophagy: Autophagic Removal of Peroxisomes

Autophagy of peroxisomes, pexophagy, is a selective degradation process of peroxisomes during which the UPS and autophagy mechanisms work in collaboration. Peroxisomes are responsible of a number of cellular functions, including fatty acid oxidation, purine metabolism and phospholipid synthesis (Wanders et al., 2016). Several peroxisomal enzymes are involved in redox regulation due to their dual functions in the generation and scavenging of reactive oxygen and nitrogen species. Therefore, peroxisome biogenesis and degradation must be tightly regulated in order to control peroxisome size, number and function (Du et al., 2015; Honsho et al., 2016). Moreover under stress conditions such as hypoxia, oxidative stress, starvation or conditions causing UPS defects, pexophagy is upregulated.

**FIGURE 8:**
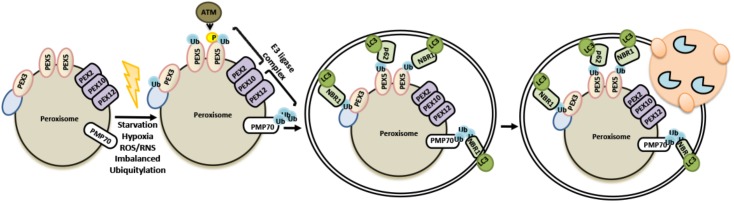
Selective removal of peroxisomes by autophagy utilizes ubiquitylation as signal.

During pexophagy, a number of peroxisomal membrane proteins, including peroxins and PMP70 become ubiquitylated (Kim et al., 2008). PEX2-PEX10-PEX12 complex serves as an E3 ligase at least for two well studied peroxisome proteins, PEX5 and PMP70. Ubiquitylation of peroxisome proteins result in the recruitment of p62 and/or NBR1 autophagy receptors, priming these organelles for autophagic degradation. For example, PEX2 overexpression or amino acid starvation activated the ubiquitylation of PEX5, and another peroxisomal membrane protein, PMP70, and led to peroxisome degradation (Sargent et al., 2016). Moreover in response to oxidative stress, ATM was recruited onto peroxisomes through physical interaction with PEX5 and promote its ubiquitylation. Inactivation of mTORC1 in a TSC2-dependent manner and stimulation of ULK1 phosphorylation by ATM, potentiated pexophagy (Zhang J. et al., 2015; Tripathi et al., 2016; Wang and Subramani, 2017). On the other hand, AAA ATPase complex (PEX1, PEX6, and PEX26) was shown to extract ubiquitylated PEX5 from peroxisomal membranes and regulate pexophagy (Carvalho et al., 2007; Okumoto et al., 2011; Law et al., 2017) (**Figure 8**). Both NBR1 and p62 were shown to be recruited onto peroxisomes during pexophagy. Yet, NBR1 was a major pexophagy receptor in a number of contexts, and p62 increased the efficiency of NBR1-dependent pexophagy through direct interaction with the latter (Deosaran et al., 2013; Zhang J. et al., 2015; Sargent et al., 2016). Altogether, these findings underline the importance of ubiquitylation for the selective degradation of peroxisomes by autophagy.

### Autophagic Removal of Ribosomes and Stress Granules

In addition to major cellular organelles, autophagy was implicated in the clearance of ribosomes. Although ribosomes can be degraded in a non-specific manner during non-selective autophagy, a special form of selective autophagy is activated under various stress conditions, and the process is called ribosomal autophagy or ribophagy. On the other hand, mRNA protein complexes that are stalled during translation form stress granules, and their clearance requires both the UPS and autophagy.

**FIGURE 9:**
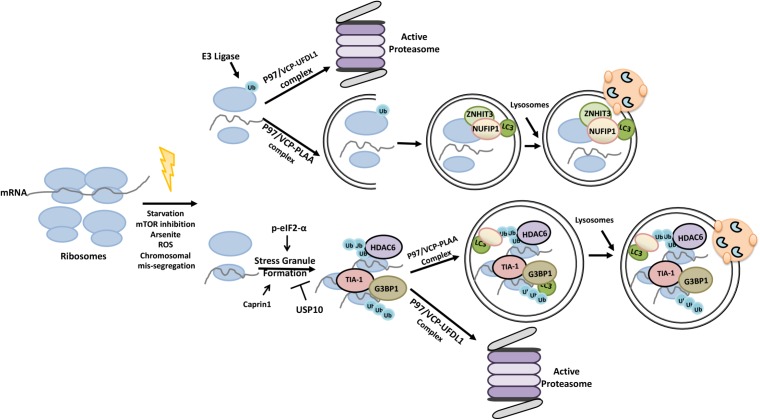
Ubiquitylation primes ribosomes and stress granules for proteasomal degradation and autophagic elimination.

Ribophagy was first described in the yeast during nutrient stress, and was shown to involve ubiquitylation of the 60S ribosome protein Rpl25 by the ubiquitin ligase Ltn1/Rkr1 (Kraft and Peter, 2008; Kraft et al., 2008; Ossareh-Nazari et al., 2014). In the mammalian system, in addition to mTOR inhibition, oxidative stress, induction of chromosomal mis-segregation, translation inhibition and stress granule formation were all shown to induce ribophagy (An and Harper, 2018). Ubiquitylation of ribosomes was observed under ER stress-inducing conditions (Higgins et al., 2015). P97/VCP that binds to ubiquitylated proteins and that functions in the delivery of these substrates to proteasome was necessary for ribophagy both in yeast and mammalian cells (Verma et al., 2013; An and Harper, 2018). Yet, individual ribosomal proteins were indeed shown to be a target of the UPS (Wyant et al., 2018). NUFIP1-ZNHIT3 proteins were identified as novel ribophagy receptors that directly connected ribosomes to LC3 and autophagy, yet whether ubiquitylation is a prerequisite for ribophagy needs to be clarified by future studies (Wyant et al., 2018) (**Figure 9**).

Stress granules are composed of actively accumulated non-translating mRNA ribonucleoprotein complexes (Protter and Parker, 2016). Proteins that accumulated in the stress granules, include stalled 40S ribosomal units and various translation initiation factors [e.g., eIF4E, eIF4G, eIF3, eIF2 and poly(A)-binding protein (PABP)] and regulators such as eIF2-α and GCN2 (Kedersha et al., 2005; Mazroui et al., 2007; Farny et al., 2009; Reineke and Lloyd, 2013). G3BP1 and TIA-1 are also among the proteins that contribute to stress granule formation (Kedersha et al., 2000; Tourrière et al., 2003; Waris et al., 2014). Moreover, an interplay between G3BP1 and Caprin1 proteins and the DUB protein USP10 was shown to regulate stress granule formation (Kedersha et al., 2016). HDAC6 protein was a component of stress granules as well (Seguin et al., 2014).

Accumulating data indicate that both the UPS and autophagy play a role in stress granüle control and elimination, and the p97/VCP protein was a key component in these processes. For example, inhibition of autophagy or p97/VCP deficiency was linked to decreased stress granule removal (Buchan et al., 2013). Co-factors of p97/VCP determined target selectivity of the protein. In this context, while the association of p97/VCP with the co-factor UFD1L led to the degradation of defective ribosomal products and dysfunctional 60S ribosomes by the UPS (Ju et al., 2008; Fujii et al., 2012; Verma et al., 2013), HDAC6 containing p97/VCP and PLAA associated granules were made a target of ribophagy (Ossareh-Nazari et al., 2010). Therefore depending on the co-factor of choice, p97/VCP has a decisive role in the choice of the degradative pathway through which ribonuclear substrates are eliminated.

### Cross Talk Between UPS and Autophagy During Endoplasmic Reticulum Stress

Endoplasmic reticulum (ER) stress is one of the conditions under which both the UPS and autophagy pathways are being activated. Abnormalities in calcium homeostasis, oxidative stress and conditions leading to protein glycosylation or folding defects etc. may result in the accumulation of misfolded and/or unfolded proteins in the ER lumen, a condition known as ER stress. ER stress might be very destructive for cells, therefore ER-specific stress response pathways such as the unfolded protein response (UPR) and the ER-associated degradation (ERAD) pathways were evolved. Both pathways are directly or indirectly connected to the UPS and autophagy.

**FIGURE 10:**
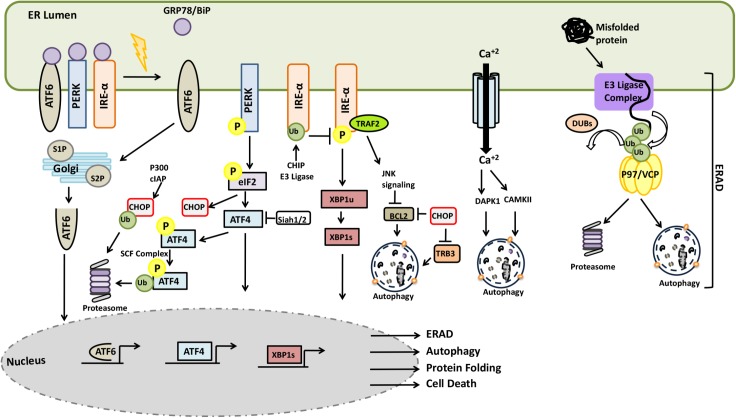
Crosstalk between the UPS and autophagy systems during ER stress and ERAD.

In mammalian cells, accumulation of unfolded proteins in the lumen of the ER result in the activation of stress responses. Following protein accumulation in the ER, the chaperone protein GRP78/BiP dissociates from the lumen-facing parts of the transmembrane proteins IRE1, ATF-6, and PERK and bind to unfolded proteins in order to assist their refolding. GRP78/BiP release triggers activation of these stress proteins (Bertolotti et al., 2000; Shen et al., 2002). PERK activation leads to the phosphorylation of the α subunit of the translation initiation factor, eIF2α, which inhibits the assembly of the 80S ribosome and cap-dependent protein synthesis, while allowing cap-independent translation of the stress response genes such as ATF4. Activation of IRE1 and ATF6 promotes transcription of other stress response genes. IRE1-mediated processing generates a splice-form of the XBP1 mRNA, resulting in the production of a transcription factor that upregulates chaperones and other relevant genes. GRP78/BiP dissociation results in the transfer of ATF6 to Golgi where cleavage of the protein by S1P and S2P proteases creates an N-terminal ATF6 fragment possessing a transcriptional activity (**Figure 10**). Due to a decrease in the protein load in the ER and an increased folding capacity, the UPR facilitates recovery from stress. In case of failure, the UPR sensitizes cells to programmed death mechanisms.

Components of the UPR were subject to active regulation by the UPS. For example, SCF component E3 ligase βTrCP was shown to lead to the ubiquitylation ATF4 following its phosphorylation (Lassot et al., 2001). On one other hand, persistent ER stress induced transcription of E3 ligase Siah1/2 following PERK-ATF4 and IRE1-XBP1 activation. On the other hand, by targeting prolyl hydroxylase PHD3, Siah1/2 was shown to regulate ATF4 hydroxylation and activity (Scortegagna et al., 2014). CHOP stability was regulated by the UPS and p300 and cIAP were responsible for CHOP ubiquitylation and degradation counterbalancing its upregulation during ER stress (Qi and Xia, 2012; Jeong et al., 2014). Another UPR component, IRE1 was identified as a ubiquitylation target of the E3 ligase CHIP during ER stress. Ubiquitylation IRE1 inhibited its phosphorylation, perturbed its interaction with TRAF2, and attenuating JNK signaling (Zhu et al., 2014). Under stress conditions, translation of XIAP, an E3 ligase protein and an inhibitor of apoptosis was downregulated in a PERK-eIF2α-dependent manner. In the same context, ATF4 may promote ubiquitylation and degradation of XIAP, leading to sensitization of cells to ER stress-related cell death (Hiramatsu et al., 2014). Conversely, activation of PERK-eIF2α axis might also show opposing effects through induction of other IAP proteins, cIAP1 and cIAP2, and counter balance cell death induction signals (Hamanaka et al., 2009).

Endoplasmic reticulum stress was shown to trigger autophagy, and ER-related stress response mechanisms were involved in the process. PERK-mediated phosphorylation of eIF2α and resulting ATF4 and CHOP activation, were associated with the transcription of genes such as ATG5, ATG12, Beclin1, ATG16L1, LC3, p62 and TSC2 activator, hence mTOR inhibitor REDD1 (Whitney et al., 2009; B’Chir et al., 2013). Moreover, CHOP downregulated BCL2 binding (Mccullough et al., 2001). TRB3, an AKT inhibitor protein, was also described as a target of CHOP (Ohoka et al., 2005). In addition, IRE1 activation resulted in the recruitment of ASK1 by the adaptor TRAF2 and the outcome was the activation of JNK and p38 kinases (Nishitoh et al., 2002). BCL2 is one of the targets of JNK, its phosphorylation by the kinase resulted in destabilization the inhibitory BCL2-Beclin1 complex, stimulating autophagy (Bassik et al., 2004). On the other hand, in its unspliced form, IRE1 splicing target XBP1, in its unspliced form was shown to target the autophagy activator FOXO1 for degradation by the UPS (Vidal et al., 2012; Xiong et al., 2012).

Endoplasmic reticulum is a major calcium store in cells, and calcium release to cytosol was observed during ER stress. In addition to problems with SERCA refill pumps and leakiness of membranes during stress, upregulation of ERO1-α by CHOP resulted in an IP3-mediated calcium release (Li et al., 2009). Calcium binding protein calmodulin senses the cytosolic increase in the concentration of the ion, and bind to calmodulin-regulated kinases such as CaMKII and DAPK1, modulating their activity. Activated CaMKII was shown to stimulate autophagy through AMPK phosphorylation and activation (Høyer-Hansen et al., 2007). In addition, calmodulin-binding and PP2A-mediated dephosphorylation was necessary for the activation of the autophagy-related kinase DAPK1 (Gozuacik et al., 2008). DAPK1 could directly phosphorylate Beclin1 on the BH3-domain, resulting in the dissociation of Beclin1 from the BCL2-Beclin1 complex and allowing it to stimulate autophagy (Zalckvar et al., 2009).

Proteins that accumulate in the ER are degraded by the ER-associated degradation (ERAD) system. ERAD mediates transport, extraction and ubiquitylation of proteins that cannot be salvaged and target them for degradation in proteasomes. In mammalian cells, ER membrane-resident complexes containing E3 ligases such as HRD1 and GP78, and other regulatory components such as EDEM1, SEL1L, ERManI, and HERP control the ERAD pathway. P97/VCP protein and its co-factors also play a role in the pathway (DeLaBarre et al., 2006; Nowis et al., 2006). Unfolded/misfolded proteins are recognized in the lumen of the ER by chaperone proteins, including BiP/GRP78 and EDEM1, and are then subsequently targeted them to the ERAD pathway. During retrotranslocation of client proteins to cytosol, ubiquitylation is followed by a p97/VCP-assisted extraction. P97/VCP also assists in the delivery of proteins to proteasomes for degradation. DUB proteins, including YOD1, USP13, USP19, and Ataxin-3 were implicated in the control of client protein ubiquitylation and ERAD substrate modulation (Zhong and Pittman, 2006; Bernardi et al., 2013; Liu Y. et al., 2014; Harada et al., 2016).

ER-associated degradation regulators and therefore ERAD might be controlled by the UPS and autophagy pathways. For example, E3 ligase Smurf1 was found to be downregulated during ER stress, resulting in the accumulation of its direct ubiquitylation target WFS, which is a stabilizer ER-related E3 ligase HRD1 (Guo et al., 2011). Smurf1 was also involved in selective bacterial autophagy (Franco et al., 2017). On the other hand, while the ERAD complex component HERP protein was degraded by the UPS (Hori et al., 2004), EDEM1 and ERManI proteins were eliminated by the autophagy machinery (Le Fourn et al., 2013; Park et al., 2014; Benyair et al., 2015). An ER-localized E3 ligase synoviolin protein was shown to ubiquitylate HERP protein and control its degradation by proteasome (Maeda et al., 2018). Yet, other ERAD-related components, EDEM1 and Derlin2 as well as ubiquitylated EDEM1 proteins colocalized with cytoplasmic aggregates and autophagy receptors p62 and NBR1, they were degraded by selective autophagy (Le Fourn et al., 2013; Park et al., 2014). ERManI, a mannosidase that is responsible for priming ER-resident glycosylated proteins for degradation, was described as an accelerator of the ERAD pathway and clearance of clients by the UPS. But, following proteasome inhibition and subsequent ER stress, ERManI colocalized with LC3 and degraded in an autophagy-dependent manner (Benyair et al., 2015).

All these findings point out to the presence of important junctions and coregulation nodes between the UPS and autophagy in the context of ER stress. Additionally, ERphagy, the autophagy of portions of the ER, was implicated in the recovery from ER stress and control of ER size, but this mechanism was so far described as a ubiquitin-independent process (Schuck et al., 2014).

### Transcriptional Mechanisms Connecting the UPS and Autophagy

Several transcription factors that are regulated by the UPS, including p53, NFκB, HIF1α, and FOXO, have been implicated in the control of autophagy. In general, these factors were shown to directly activate transcription of key autophagy genes under stress conditions. Some autophagy proteins such as LC3 are consumed in the lysosome following delivery, and during prolonged stress, cellular levels of these proteins are sustained by mechanisms, including transcription. On the other hand, regulation of the transcriptional activity NRF2 involves a special crosstalk between the two systems. In this section, we will summarize molecular details of transcription regulation by the UPS and autophagy.

P53, a guardian of the genome, is one of the well-known transcriptonal regulators that has a dual role in autophagy depending on its intracellular localization. In the absence of stress, cellular p53 levels are controlled by the E3 ligase HDM2/MDM2 and the UPS. Under stress conditions, p14/p19/ARF protein binds, sequesters and inactivates HDM2/MDM2, stabilizing p53. Accumulating p53 protein activates transcription of several stress- and death-related genes, including autophagy-related genes *PRKAB1, PRKAB2, TSC2, ATG2, ATG4, ATG7, ATG10,ULK1, BNIP3, DRAM1*, and *SESN2* (Crighton et al., 2006; Feng et al., 2007; Budanov and Karin, 2009; Kenzelmann Broz et al., 2013). On the other hand, a cytosolic form of p53 was shown to inhibit AMPK and activate the mTOR pathway. In this context, non-genotoxic stress by autophagy-inducing agents such as rapamycin, tunicamycin and nutrient deprivation favored HMD2/MDM2-dependent p53 degradation by the UPS (Tasdemir et al., 2008a,b). Interestingly, HMD2/MDM2 stability and activity were also regulated by E3 ligases SMURF1/2 which in turn affected the stability of p53. SMURF1/2-mediated ubiquitylation was shown to increase MDM2-MDMX heterodimerization, decreasing autoubiquitylation of MDM2, therefore stabilized the protein (Nie et al., 2010). Additionally, another E3 ligase, NEDD4-1 was shown to control MDM2 stability and p53 activation (Xu et al., 2015). In addition to MDM2, another E3 ligase, PIRH2, was able to ubiquitylate p53 to control its cellular stability (Shloush et al., 2011).

NF-κB is a well studied transcriptional regulator of autophagy. As a result of its association with IκB, NF-κB is found in an inactive state in the cytosol. In response to agonists, IκB was reported to be ubiquitylated and subsequently degraded by the UPS. Regulation of NF-κB by external signals involved phosphorylation of IκB by upstream kinases of the IKK complex (IKKα, IKKβ, and IKKγ/NEMO). Phosphorylated IκB recruits the E3 ligase SCF-βTRCP, followed by its degradation in the proteasome (Orian et al., 2000). After IκB degradation, NF-κB was then free to migrate to the nucleus of the cell, and induce transcription of target genes, including Beclin1 and p62, and induce autophagy (Copetti et al., 2009; Ling et al., 2012).

Another level of regulation involved TNF-α receptor-associated protein complexes. Binding of TNF-α to TNFR1 led to the recruitment of TRADD and RIPK1 to the receptor, promoting TRAF- and cIAP-mediated K63 and/or K11 linked ubiquitylation of the RIPK1. Ubiquitylated RIPK1 could recruit NEMO and TAB-TAK1 complex for IKK activation and hence NF-κB stimulation. Additionally, RIPK1 could also be modified by A20 through addition of K48-linked poly-ubiquitin chains, sending the kinase for proteasomal degradation (Kravtsova-ivantsiv et al., 2015).

However, in some contexts, TNF-α-induced NF-κB activation was reported to inhibit autophagy (Djavaheri-Mergny et al., 2006). TNF-α-induced activation of IKKα or IKKβ could stimulate phosphorylation of TSC1/2 and activate mTOR, leading to a similar inhibitory outcome (Lee et al., 2007; Dan and Baldwin, 2008). Furthermore in some contexts, RIPK1 silencing activated autophagy under both basal and stress conditions (Yonekawa et al., 2015). On the other hand, RIPK1 itself was reported to be a target of p62-mediated selective autophagy (Goodall et al., 2016). Moreover, autophagy was responsible for the degradation of NF-κB activator NIK and IKK complex subunits, indicating the presence of a tight cross-regulation of the NF-κB pathway by the UPS and autophagy (Qing et al., 2007).

Another transcription factor that was controlling the autophagic outcome was HIF1α. Hypoxia induced HIF1α transcriptionally regulated various hypoxia response genes, including *GLUT1* (Chen et al., 2001), *NOX2* (Yuan et al., 2011), and *PDK1* (Kim et al., 2006) as well as autophagy genes, including *BNIP3, BNIP3L, ATG5*, and *BECN1* to stimulate autophagy, mitophagy, and pexophagy (Zhang et al., 2008; Bellot et al., 2009; Walter et al., 2014). HIF1α itself was regulated in a UPS-dependent manner. Under normoxia, hydroxylation of HIF1α specific prolyl hydroxylases (PHDs) hydroxylated HIF1α (Jaakkola et al., 2014) served as a recognition signal for UbcH5, an E2 enzyme and von Hippel-Lindau protein (the pVHL), E3 ligase complex containing Elongin B and C, Cullin-2, and Rbx1 allowing K48 linked ubiquitination of HIF1α and its proteasomal degradation (Ohh et al., 2000; Lee et al., 2015). In contrast, during hypoxia, PHDs were inhibited and HIF1α stabilized. SCF E3 ligase complex was also a regulator of HIF1α stability in response to GSK3β-mediated phosphorylation of the protein (Cassavaugh et al., 2011; Flugel et al., 2012). Another E3 ligase facilitating HIF1α degradation was HAF (also known as SART1_800_). Unlike pVHL, HAF-mediated ubiquitylation of HIF1α was not depending on the oxygen levels, providing an alternative HIF1α regulation mechanism (Koh et al., 2008). Stability of PHD proteins were also controlled by the UPS. For example, SIAH1/2 was shown to direct PHDs for proteasomal degradation under hypoxic stress (Nakayama et al., 2004). Moreover several DUBs were implicated in HIF1α regulation, including USP20 (Li et al., 2002b), USP28 (Flugel et al., 2012), and USP33 (Li et al., 2002a).

FOXO family of transcription factors (FOXOs) were associated with various cellular pathways, including autophagy (Zhao et al., 2007). The activity of FOXOs were regulated by their phosphorylation status and following activation, FOXOs translocated to the nucleus and triggered the expression of a number of genes associated with different stages of the autophagy pathway, including *ATG4, ATG12, BECN1, ULK1, PIK3C3, MAP1LC3*, and *GABARAP* (Mammucari et al., 2007; Zhao et al., 2007; Sanchez et al., 2012). There are several connections between FOXOs and autophagy. Activation of the AKT pathway inhibited FOXO3 activity, led to a decrease in LC3 and BNIP3 expression, therefore blocked autophagy (Stitt et al., 2004; Mammucari et al., 2007). On the other hand, AMPK activation led to the phosphorylation of FOXO3a and ULK1, inducing *MAP1LC3, GABARAP*, and *BECN1* expression and subsequent autophagy activation (Sanchez et al., 2012). Another FOXO family protein FOXK1/2, a negative regulator of FOXO3, was associated with a decrease in autophagy by removing Sin3A/HDAC complex from histone H4 to diminish its acetylation. In this context, nuclear localization of FOXK1/2 was mTOR-dependent and showed an inhibitory effect on autophagy gene expression under basal conditions (Bowman et al., 2014). Moreover, JNK deficiency in neurons increased autophagic activity through FOXO1-mediated BNIP3 upregulation and Beclin1 disassociation from BCL-XL (Xu et al., 2011). Another example of a link between FOXOs autophagy involved ATG14. Liver specific knockout of FOXOs resulted in the downregulation of ATG14 and this event was associated with high levels of triglycerides in the liver and serum of mice (Xiong et al., 2012). Additionally, GATA-1 shown to directly regulate FOXO3-mediated activation of LC3 genes to facilitate autophagic activity (Kang et al., 2012).

Phosphorylation of FOXO proteins by various protein kinases, including AKT, IKK, and ERK, affected their ubiquitylation by E3 ligases and their stability (Huang and Tindall, 2011). For instance, AKT-mediated phosphorylation of FOXO1 provided a signal for its recognition by the SKP protein, an SCF E3 ligase complex component, followed by FOXO1 ubiquitylation and degradation (Huang et al., 2005). COP1 was also identified as an E3 ligase that regulated FOXO protein stability. COP1 ubiquitylated FOXO1 and promoted its proteasomal degradation. This type of regulation might be important in the glucose metabolism of hepatocytes, and possibly in autophagy modulation under this conditions (Kato et al., 2008). Another FOXO regulating E3 ligase was MDM2 that was reported to be responsible for FOXO1 and FOXO3A ubiquitylation and degradation (Fu et al., 2009). MDM2-mediated ubiquitylation was activated by the phosphorylation of FOXOs by AKT. Due to its role in p53 regulation, MDM2 could be part of a more complex regulatory mechanism which might link the UPS, transcriptional regulation and autophagic activity.

NRF2-KEAP1-P62 pathway was defined as another major oxidative stress response mechanism involving an interplay between the UPS and autophagy. NRF2 is a transcription factor, and when activated, is upregulated antioxidant and metabolic enzymes, including *TXNRD1* (Suvorova et al., 2009), *HMOX1* (Reichard et al., 2007), *GPX2* (Banning et al., 2005), *GBE1, PHK1* (Banning et al., 2005), and downregulated proinflammation-related genes such as *IL6, IL1B* (Kobayashi et al., 2016). KEAP1 is an adaptor protein of the E3 ligase Cullin-3 and plays a role in substrate recognition. Under normal conditions, transcription factor NRF2 was found in association with KEAP1-Cullin-3 E3 ligase complex, that catalyzed its ubiquitylation, rendering it a substrate for proteasomal elimination by selective autophagy (Ishimura et al., 2014). Competition resulted in the migration of free NRF2 to the nucleus and transactivation of stress-related cytoprotective genes (Kobayashi et al., 2004; Komatsu et al., 2010). Additionally, the NRF2–KEAP1 pathway provides a positive feedback loop for autophagy. P62 was characterized as a direct transcriptional target of activated NRF2 (Jain et al., 2010). Moreover, KEAP1 regulation by p62 was modulated by the E3 ligase TRIM21. NRF2 activation was negatively affected by TRIM21-mediated K63-linked ubiquitylation of p62 (Pan et al., 2016).

### Autophagy-UPS Crosstalk in Diseases

Crosstalk between autophagy and the UPS may change character under disease conditions, contribute to the pathogenesis of diseases and even affect their outcome. Degenerative diseases and cancer are examples of diseases that illustrate the interplay between the UPS and autophagy in the clearance of misfolded abnormal proteins (Juenemann et al., 2013).

For example, Huntington Disease is caused by poly-glutamine extensions in a protein called Huntingtin (Htt), leading to abnormal organization and eventual aggregation of the protein. Htt protein was shown to be ubiquitylated via K48- or K63-linked ubiquitin chains (Bhat et al., 2014). Mutant Htt clearance depended on both the UPS and autophagy in different experimental settings. Mutant Htt aggregates were largely cleared by K63-dependent autophagy mechanisms (Renna et al., 2010; Menzies et al., 2015). On the other hand, overexpression of K48-specific E3 ligase Ube3a, resulted in a UPS-dependent degradation of mutant proteins. Yet, cellular levels of E3 ligase was shown to decline in an age-dependent manner. Therefore, in elderly people, accumulation of K63-linked polyubiquitylated proteins might tip the balance toward clearance of protein aggregates by autophagy. A similar UPS switch was also observed in a CHIP-dependent manner (Jana et al., 2005; Bhat et al., 2014).

Another example involves the ERAD protein p97/VCP. Mutant forms of the protein were associated with a rare syndrome that mainly affects muscles, bones and the brain (Inclusion Body Myopathy with the Paget’s Disease of Bone and frontotemporal Dementia, IBMPFD). Moreover, p97/VCP mutations were detected in a fraction of patients suffering from familial forms of Parkinson’s Disease or from Amyotrophic Lateral Sclerosis (ALS) (Johnson et al., 2010). As mentioned in the previous sections, p97/VCP is important for the extraction of misfolded ER proteins as well as their delivery to proteasomes. Moreover, p97/VCP was proposed to play a role in autophagosome maturation and autolysosome formation (Tresse et al., 2010). We recently showed that some of the disease-related mutations of p97/VCP (namely P137L and G157R) resulted in the aggregation of the protein itself. Mutant p97/VCP proteins formed complexes with wild-type counterparts and led to further accumulation of ubiquitylated proteins upon ER stress, indicating that the ERAD system was negatively affected by the mutant (Bayraktar et al., 2016). Indeed, ERAD co-factor and ubiquitin binding capacity of the mutant p97/VCP was decreased (Erzurumlu et al., 2013). Yet, autophagy was still functional under these conditions, and could significantly eliminate these aggregates (Bayraktar et al., 2016). Therefore, preferential elimination of mutant proteins by autophagy might tip the balance in favor of wild-type proteins and restore disease-related loss of cellular functions including UPS-related mechanisms.

The role of the crosstalk between the two systems is also prominent in the cancer context. For example, the P53-regulated and cancer-related protein EI24, was introduced as a critical link between the UPS and autophagy (Devkota et al., 2012). EI24 controlled the stability of E3 ligases TRIM41, TRIM2, and TRIM28 by the regulation of their autophagic degradation (Devkota et al., 2016; Nam et al., 2017). Cellular levels of other E3 ligases, namely MDM2 and TRAF2, were also regulated by EI24-controlled degradation, modulating p53 and mTOR pathways, respectively, and influencing cancer formation and progression (Devkota et al., 2016).

Deregulation and/or mutations of proteins that function in the autophagy and/or the UPS were observed in some cancer types, resulting in the modification of individual pathways and possibly affecting the crosstalk between the two systems. Changes include, modulation of levels of E3 ligases such as MDM2 (Haupt et al., 2017), SMURF1 (Fukunaga et al., 2008; Kwon et al., 2013), SCF components (e.g., βTrCP), point mutations of NEDD4 (Amodio et al., 2010), COP1 (Marine, 2012), FBXW7 (Korphaisarn et al., 2017), and mutations in autophagy related proteins Beclin1 (Laddha et al., 2014), LKB1 (Ji et al., 2007), ATG5 (Takamura et al., 2011), ATG4C (Marino et al., 2007) as well as deletions of genes of proteins, such as Beclin1 (Liang et al., 1999; Qu et al., 2003), AMPK (Li et al., 2015) and UVRAG (He et al., 2015). Under these circumstances, dynamic and complex changes in the regulation of the degradative pathways should have dramatic effects that contribute to cancer-related alterations in the proteomic landscape of cells.

Autophagy-UPS crosstalk emerges as a critical factor that determines the success of disease treatment, chemotherapy is one striking example. For instance, proteasome inhibition by the chemotherapy agent bortezomib resulted in the accumulation of misfolded proteins and induced compensatory autophagy in cancer cells (Obeng et al., 2006). Under these circumstances, autophagic activity protected cancer cells from bortezomib-induced cell death, and inhibition of autophagy improved the outcome of chemotherapy. These dual autophagy-UPS targeting approaches also gave promising results in clinical trials (Vogl et al., 2014).

Several companies are now developing drugs that modulate the UPS or autophagy [for example, (Huang and Dixit, 2016)]. Concepts and data that were discussed above and elsewhere indicate that, depending on the disease type and treatment strategy, the crosstalk between the UPS and autophagy should definitely be taken into account in these efforts.

## Conclusion and Perspectives

Autophagy and the ubiquitin proteasome systems are major degradation systems in mammalian cells that allow recycling of cellular contents ranging from soluble proteins to intracellular organelles. Although their mode of action and their requirements for substrate recognition are different, there are several overlaps and interconnections between the UPS and autophagy pathways.

A prominent component of the crosstalk is the ubiquitin protein itself and ubiquitylation. Indeed, ubiquitin is a common signal for both the UPS and autophagy. It was proposed that, ubiquitin chain type could determine the pathway of choice for protein degradation. K48-linked ubiquitylation was introduced to be a signal for the UPS, whereas K63-linked ubiquitylation directed proteins for autophagosomal degradation (Herhaus and Dikic, 2015). Yet, a number of independent studies provided evidence that both ubiquitylation types could lead to autophagic degradation of substrates (Wandel et al., 2017). Moreover, recent studies underline the importance of ubiquitin phosphorylation as an event that increased the affinity of autophagy receptors for their targets during selective autophagy (Kane et al., 2014; Koyano et al., 2014). Additionally, non-ubiquitin modifications (e.g., acetylation, sumoylation, neddylation etc.) were shown to affect protein degradation as well (Hwang and Lee, 2017). Therefore, a barcode of ubiquitin and other modifications seem to prime proteins for one or the other degradation pathway and determine their fate. As another level of regulation, deconjugating enzymes such as DUBs may counteract or redirect proteins for different degradation systems.

E3 ligases emerged as important components of the UPS-autophagy switches. For example, Cullin-3 (Pintard et al., 2004), SMURF1 (Ebisawa et al., 2001), MDM2 (Shi and Gu, 2012) E3 ligases directed proteins to degradation by the UPS, whereas the role of Parkin (Chan et al., 2011), LRSAM1 (Huett et al., 2012), and CHIP (Shin et al., 2005) in priming proteins for autophagic degradation was observed in several studies. On the other hand, the same E3 ligase that might be able to generate different ubiquitin linkages under different conditions and on different substrates (Chan et al., 2011), the switch between degradative pathways being controlled by specific E3 ligase adaptors, post-translational modifications on target proteins as well as other unknown factors. A prominent example is the Parkin protein. During mitophagy although some of the proteins that are ubiquitylated by Parkin are degraded, other ubiquitylated proteins contribute to mitochondrial clustering and recognition by autophagy receptors. To date, factors or modifications that determine the substrate selectivity of Parkin are unknown.

Another example of UPS-autophagy switch involves the p97/VCP protein. While binding of the co-factor PLAA to p97/VCP resulted in the autophagic degradation of ubiquitylated clients of the protein, binding of UFDL1 as a co-factor favored degradation by the UPS. Moreover, p97/VCP was also associated with aggregate formation in collaboration with some autophagy receptors.

Signaling switches involved in the regulated activation of one or the other system was shown to modify cellular responses to stress. For example, NRF2 degradation by the UPS was controlled through p62-mediated KEAP1 elimination by autophagy (Jain et al., 2010). Prevention of HIF1α degradation by the UPS, resulted in the expression of stress response genes, including autophagy genes, led to autophagy activation. In another example, the UPS activity was required for NF-κB activation and NF-κB-mediated autophagy gene upregulation. Yet, autophagic degradation of NF-κB activators NIK and IKKs provided a negative feedback loop in the control in this context (Qing et al., 2007). Therefore, modification of cellular signaling pathways by degradative systems might modulate upstream signals that control autophagy and/or the UPS, and affect their activation and amplitude.

Degradation of the components or regulators of one system by the other system was also reported. For example, proteasomes were defined as substrates of selective autophagy (Marshall et al., 2015). Conversely, various autophagy proteins were ubiquitylated and degraded by the UPS in a regulated manner. Therefore, checks and balances between the two systems exist, and these control mechanisms possibly allow remodeling of the cellular proteome under different conditions.

Compensation mechanisms are also operational between the two systems. Inhibition of the UPS generally upregulated autophagy, whereas failures in the autophagy system were associated with increased UPS activity, although inefficient compensation and failure in both systems were also observed under certain conditions (Korolchuk et al., 2009a,b). Moreover, alternative protein degradation pathways, such as CMA and microautophagy might come into play under these conditions as well. Nevertheless, depending on the character of the target to be degraded, compensation mechanisms were less or more effective. For example, large aggregates and whole organelles should be cleared by autophagy, but defective ribosomal products that could not be accumulated in stress granules were shown to be directed for proteasomal degradation. Therefore for cellular homeostasis and for proper functioning of cells, ideally both systems should be fully operational.

Data obtained so far demonstrate that crosstalk and communication between autophagy and the UPS generally rely on non-specialized and even indirect links. Yet, there might exist so far unknown specialized proteins providing coordination and co-regulation of the two systems. Furthermore, regulation through direct protein-protein interactions between known system components is another possibility. Therefore, dedicated communication proteins or pathways between the degradation mechanisms may be present, allowing better and faster coordination in case of need. Further studies are required to unveil the nature of these putative proteins, interactions and pathways.

An emerging theme in the regulation and coordination of autophagy and the UPS involves non-coding RNAs and their intricate networks. A growing list of microRNAs as well as long non-coding RNAs were implicated in the control of autophagy (Tekirdag et al., 2016) as well as the UPS (Wu and Pfeffer, 2016; Chang et al., 2018). MicroRNAs have the advantage of affecting the level of multiple proteins at once, and they are able to rapidly reshape cellular signaling mechanisms and pathways. Therefore, non-coding RNA networks possibly contribute to the co-regulation of these degradative systems. Intriguingly, deregulation of non-coding RNA levels contribute to the progression of diseases such as cancer. Future studies on non-coding RNAs will reveal their relevance in the autophagy-UPS crosstalk under physiological and pathological conditions.

Overall, coordination, interconnection and crosstalk mechanisms between the UPS and autophagy exist at various levels. In addition to ubiquitin and ubiquitylation, several proteins and signaling pathways were implicated in the communication and mutual regulation of the two systems. Considering the importance of protein catabolism for cellular and organismal homeostasis and health, a better understanding of individual systems as well as the interconnections and crosstalks between them will be most rewarding from both a basic science perspective and with regards to clinical management of diseases involving protein quality control problems.

## Author Contributions

NK and DG wrote the manuscript and did critical reading. NK prepared the illustrations in the manuscript.

## Conflict of Interest Statement

The authors declare that the research was conducted in the absence of any commercial or financial relationships that could be construed as a potential conflict of interest.
